# Comparison of individual and pooled sampling methods for estimation of *Vairimorpha* (*Nosema*) spp. levels in experimentally infected honey bee colonies

**DOI:** 10.1177/10406387231194620

**Published:** 2023-08-28

**Authors:** Sarah Biganski, Tessa Lester, Oleksii Obshta, Midhun S. Jose, Jenna M. Thebeau, Fatima Masood, Marina C. B. Silva, Marcelo P. Camilli, Muhammad F. Raza, Michael W. Zabrodski, Ivanna Kozii, Roman Koziy, Igor Moshynskyy, Elemir Simko, Sarah C. Wood

**Affiliations:** Departments of Veterinary Pathology, University of Saskatchewan, Saskatoon, SK, Canada; Departments of Veterinary Pathology, University of Saskatchewan, Saskatoon, SK, Canada; Departments of Veterinary Pathology, University of Saskatchewan, Saskatoon, SK, Canada; Departments of Veterinary Pathology, University of Saskatchewan, Saskatoon, SK, Canada; Departments of Veterinary Pathology, University of Saskatchewan, Saskatoon, SK, Canada; Veterinary Microbiology, University of Saskatchewan, Saskatoon, SK, Canada; Departments of Veterinary Pathology, University of Saskatchewan, Saskatoon, SK, Canada; Departments of Veterinary Pathology, University of Saskatchewan, Saskatoon, SK, Canada; Departments of Veterinary Pathology, University of Saskatchewan, Saskatoon, SK, Canada; Western College of Veterinary Medicine, and Prairie Diagnostic Services, University of Saskatchewan, Saskatoon, SK, Canada; Western College of Veterinary Medicine, and Prairie Diagnostic Services, University of Saskatchewan, Saskatoon, SK, Canada; Western College of Veterinary Medicine, and Prairie Diagnostic Services, University of Saskatchewan, Saskatoon, SK, Canada; Departments of Veterinary Pathology, University of Saskatchewan, Saskatoon, SK, Canada; Departments of Veterinary Pathology, University of Saskatchewan, Saskatoon, SK, Canada; Departments of Veterinary Pathology, University of Saskatchewan, Saskatoon, SK, Canada

**Keywords:** colony infection, honey bees, sampling methods, *Vairimorpha apis*, *Vairimorpha ceranae*

## Abstract

The microsporidian pathogens *Vairimorpha apis* and *V. ceranae* are known to cause intestinal infection in honey bees and are associated with decreased colony productivity and colony loss. The widely accepted method for determining *Vairimorpha* colony infection level for risk assessment and antibiotic treatment is based on spore counts of 60 pooled worker bees using light microscopy. Given that honey bee colonies consist of as many as 1,000 times more individuals, the number of bees collected for *Vairimorpha* detection may significantly impact the estimated colony infection level, especially in the case of uneven distribution of high- and low-infected individuals within a hive. Hence, we compared the frequency and severity of *Vairimorpha* infection in individual bees to pooled samples of 60, 120, and 180 bees, as well as compared the *Vairimorpha* spp. prevalence in pooled samples of 60 and 180 bees. Overall, we did not find significant differences in spore counts in pooled samples containing incremental numbers of bees, although we observed that, in less-infected colonies, a low frequency of highly infected individuals influenced the estimated colony infection level. Moreover, *Vairimorpha* spp. prevalence did not differ significantly among the pooled bee samples tested. Increasing the number of pooled bees from the recommended 60 bees to 180 bees did not yield a more accurate representation of colony infection level for highly infected colonies, but the clinical importance of a low frequency of highly infected individuals in less-infected colonies needs to be addressed in future studies.

The Western honey bee (*Apis mellifera*) plays a crucial economic and ecologic role in providing pollination services for agriculture and global food production.^17^ However, managed honey bees are faced with a large variety of biotic and abiotic stressors such as the fungal pathogens *Vairimorpha apis* and *V. ceranae* (*Nosematidae*; formerly *Nosema apis* and *N. ceranae*^26^), which cause severe and sometimes fatal disease in adult bees.^13^ Clinical signs of vairimorphosis (nosemosis) include dysentery with nutritional stress and malnutrition, shortened lifespan, impaired flight ability, and disorientation in individual forager bees in correlation with reduced colony productivity as a result of poor foraging performance and brood care.^7,20,25^ At the colony level, *Vairimorpha* spp. infection weakens colonies and increases the risk of overwinter mortality, accompanied by high economic losses for the beekeeping industry.^3,4,10,11^ The widely accepted method for estimation of *Vairimorpha* spp. infection at the colony level is to collect 60 forager bees from the hive entrance in spring and fall, macerate the bees to extract intestinal contents, followed by a microscopic spore count in a hemocytometer to estimate the spore load per bee.^2,16,21^ The total number of bees in a colony ranges from ~20,000 during early spring to ~60,000 during late summer, with 30% of the oldest bees in a colony being foragers, and reportedly having the highest spore loads in a colony.^21,27^ Considering that only a small portion of bees (0.1–0.3%) is used for estimation of the colony infection level, the true parasite burden and the distribution of infection levels of individual bees inside a colony may not be reliably reflected with a sample size of 60 foragers.^14,16,21^ *Vairimorpha* spp. surveillance of our research apiaries (~200 hives) in the summer of 2021 revealed highly variable spore counts and *Vairimorpha* spp. composition in sequential samples (containing 60 bees) from the same hive within one day and on the following days (Suppl. Table 1), which is in strong concordance with another report of the variation in *Vairimorpha* species prevalence after subsequential sampling.^2^

To evaluate the effect of sampling methods on estimation of *Vairimorpha* colony infection level, we infected colonies with ~50:50 mixture of *V. apis* and *V. ceranae* in the fall and performed the following analysis during the following spring: 1) compared the average number of spores per bee determined in incremental, pooled samples of bees; 2) counted spores in individual bees to determine the frequency of severely versus mildly infected bees, and 3) compared the ratio of *V. apis* to *V. ceranae* in individual bees and in incremental, pooled samples of bees. A pooled sample can consist of a low number of highly infected bees or an equal distribution of moderately infected bees, resulting in a similar calculated spore count per bee.^16^ Accordingly, we analyzed the frequency of spore levels in individual bees and correlated it to pooled samples to find a reliable number of bees to represent the colony infection level (objectives 1, 2). Moreover, single bees can be infected with one or both *Vairimorpha* species,^22^ so we determined the species composition in pooled samples with quantitative real-time PCR (qPCR) to correlate the species ratio with sample size (objective 3).

## Materials and methods

### Experimental colony preparation and *Vairimorpha* infection

**Figure 1.**
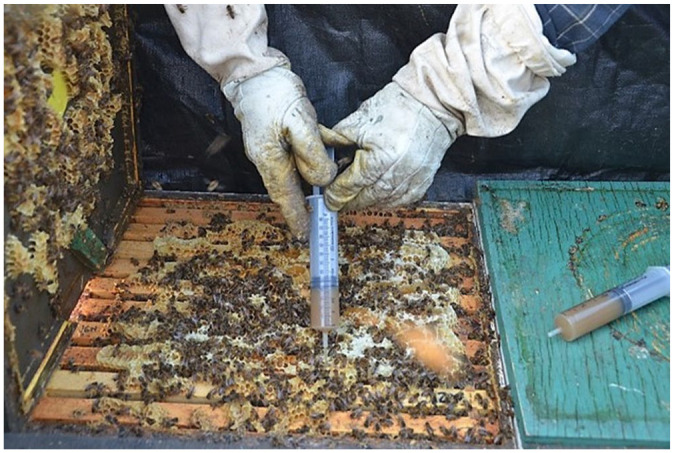
Experimental inoculation of a honey bee colony with *Vairimorpha* suspension.

In September 2021, ~80 honey bee colonies at 2 research apiaries near Saskatoon, SK, Canada were examined for *Vairimorpha* infection by microscopy and qPCR (60 bees per colony), and 21 colonies were identified as not infected. All colonies were treated with label dosages of oxytetracycline and amitraz, for control of American foulbrood and *Varroa* mites, respectively, per our region’s standard beekeeping practices. In October 2021, after colonies were wrapped for outdoor overwintering, we randomly assigned the 21 *Vairimorpha*-free colonies into 3 experimental groups (control, low-dose, high-dose), with 7 colonies per group. Colonies assigned to the low- and high-dose groups were inoculated with 90 × 10^6^ and 900 × 10^6^ *Vairimorpha* spores, respectively (90 mL of homogenate containing 10^6^ or 10^7^ spores/mL); colonies in the control group were administered 90 mL of a sucrose solution. *Vairimorpha* spores for the inoculum were obtained from ~1,000 bees from a donor colony that was determined by qPCR of 60 bees to be infected with 50% of both *Vairimorpha* species.^27^ To prepare the inoculum, we crushed the bees in a blender with NH_4_Cl to avoid spore germination and mixed the spores with 2:1 (w/v) sucrose syrup to the final concentration, having 1:1 (w/v) sucrose syrup in the final suspension.^12^ We applied the spore suspension in the inter-frame spaces directly on the bee cluster of the top super (box) with a syringe (Fig. 1). The colonies that we used had never been treated with fumagillin, either in advance of or during our experiment.

### Colony strength, and *Varroa* infestation assessment

As an estimate of colony strength, the cluster size of the top super was analyzed from images taken with a digital camera (16.2 megapixel Nikon D7000; Minato) in October 2021, before inoculation, when colonies were already wrapped for outdoor overwintering, and again in March 2022, coinciding with the first visual overwinter survival assessment.^23^ We estimated the cluster size by counting the number of inter-frame spaces in the top super occupied by adult bees to the nearest 0.25.^23^ *Varroa* mite infestation per colony was analyzed in March 2022 using the alcohol wash technique.^6,18^

### Colony *Vairimorpha* assessment

We collected ~300 bees with a vacuum from the lid of the top super in March, April, and May 2022 from all live colonies. Bees were divided into subsamples of 3 × 60 bees and 30 individual bees. Single bees were each macerated in 500 μL of sterile dH_2_O, and the 3 subsamples containing 60 bees were each homogenized with 60 mL of sterile dH_2_O. To generate pooled samples of 120 and 180 bees, we combined 400 μL of two 60-bee subsamples (= 120 pooled bees) and 400 μL of three 60-bee samples (= 180 pooled bees). *Vairimorpha* spore loads per bee were determined by evaluation of a 10-μL aliquot of each individual or 60-, 120-, or 180-bee sample using a hemocytometer and light microscope.^12^ Additionally, genomic DNA was extracted from the 60- and 180-bee samples (Blood and tissue kit; Qiagen), and *Vairimorpha* species composition was determined by qPCR with species-specific primer–probe combinations based on the small ribosomal subunit rDNA.^27^ The Ct values from qPCR were transformed to spore numbers using the slope function from duplicate standard curves prepared from 10-fold diluted spores (10^7^–10 spores) of each species.

### Statistical analysis

All statistical tests were conducted with R v.4.2.2 (https://www.r-project.org/) and an α = 0.05 significance level. Normality was assessed with the Shapiro–Wilk test. The number of surviving colonies was compared by treatment group and sampling time using the Pearson χ^2^ test. *Vairimorpha* spore counts and species prevalence were compared among pooled samples using repeated measures one-way ANOVA with a post hoc paired *t*-test and Wilcoxon matched-pairs signed rank test, respectively. The effect of treatment and sample size on species prevalence was determined by 2-way ANOVA. One-way ANOVA or the Kruskal–Wallis test were used to analyze the interaction effect of sampling time and treatment on cluster size and *Varroa* infestation. The correlation between colony infection level and frequency of infection severity or intensity (non-infected to highly infected) or *Vairimorpha* spp. prevalence was assessed with the Pearson correlation.

## Results

### Colony overwinter survival, strength, and *Varroa* infestation level

**Figure 2.**
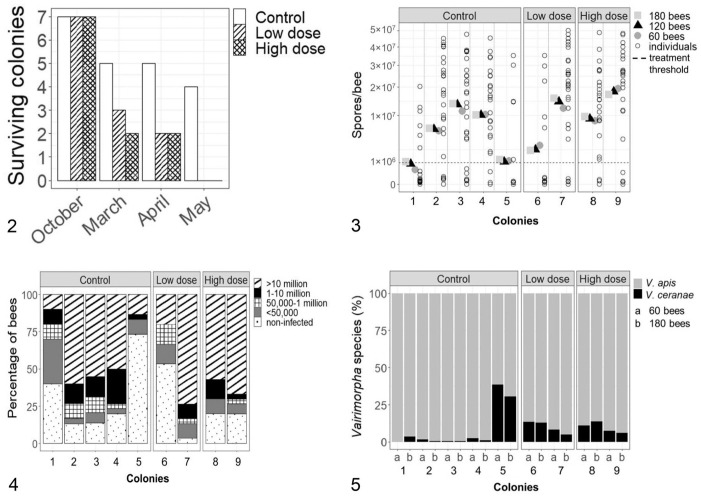
Colony overwinter survival of control and *Vairimorpha* spp.–infected colonies over time. **Figure 3.**Comparison of *Vairimorpha* spore counts in 30 individual bees and pooled samples containing 60, 120, and 180 bees collected in April 2022 from control (1–5), low-dose (6, 7) and high-dose (8, 9) colonies. **Figure 4.** Percentage of bees with different *Vairimorpha* infection intensities in control (1–5), low-dose (6, 7), and high-dose (8, 9) colonies. **Figure 5.** Proportion of *Vairimorpha apis* and *V. ceranae* in samples from control (1–5), low-dose (6, 7), and high-dose (8, 9) colonies. The *V. apis* and *V. ceranae* level was averaged for the qPCR results from 60 (a) and 180 (b) pooled bees.

Of 21 colonies, 10 survived until March 2022, with a statistically nonsignificant dose-response in colony survival (Fig. 2; χ^2^ = 6.5243, df = 6, *p* = 0.3671). Surviving colonies in all groups had significantly decreased colony strength from October 2021 to March 2022 (F_(1,36)_ = 113.247, *p* < 0.0001), but the colony strength did not differ significantly among the control and infected groups at each time (F_(2,36)_ = 1.071, *p* = 0.353; Table 1). In March 2022, the *Varroa* infestation did not differ significantly among treatment groups (F_(2,7)_ = 1.938, *p* = 0.214; Table 1).

**Table 1.** Cluster size and percent *Varroa* infestation in surviving control and *Vairimorpha* spp.–infected colonies.

	Mean cluster size, inter-frame spaces covered with bees		Significance	Mean *Varroa* infestation, x̄% ± SD
Date	2021-Oct-19	2022-Mar-23		2022-Mar-23
Control	9.4	3.7	*	2.62 ± 3.05
Low dose	9.0	3.3	*	4.45 ± 1.89
High dose	8.6	3.1	*	7.82 ± 5.07

* Significant difference (*p* < 0.05) in cluster size from October 2021 to March 2022.

### Comparison of *Vairimorpha* spore counts in pooled vs. individual samples

There was a significant (F_(2,33)_ = 3.754, *p* = 0.034) dose-response in *Vairimorpha* infection level in April 2022, with mean spore counts in control, low-dose, and high-dose groups of 7.3 ± 5.7 × 10^6^, 9.7 ± 9.2 × 10^6^, and 14.2 ± 5.9 × 10^6^ spores/bee, respectively, based on the mean spore counts in 60, 120, and 180 bees. The mean spore count per bee in 30 individually analyzed bees, and 60, 120, and 180 pooled bees was not statistically different within each treatment group (control F_(1.1,4.4)_ = 5.505, *p* = 0.0717; low-dose F_(1,1)_ = 2.019, *p* = 0.3904; high-dose F_(1,1)_ = 1.324, *p* = 0.4555), nor among the treatment groups (F_(3,24)_ = 0.846, *p* = 0.4823), and the interaction of treatment and sampling method (pooled sampling) was nonsignificant (F_(6,24)_ = 0.033, *p* = 0.9998; Fig. 3).

### Comparison of *Vairimorpha* spore counts from pooled bee samples with the frequency of *Vairimorpha* infection intensity in individual bees

An increase in mean spores per bee based on pooled sampling (Fig. 3) was correlated significantly with a decrease in the percentage of non-infected individual bees (*r* = –0.7975, *p* = 0.01; Fig. 4), and also with an increase in the percentage of highly infected individual bees (*r* = 0.904, *p* = 0.0008). Colonies with similar mean *Vairimorpha* spore loads per bee based on a pooled sample also had a comparable distribution of individual bees with different infection intensities (Figs. 3, 4). For example, colonies 1, 5, and 6 had the lowest mean spores per bee based on pooled samples (Fig. 3) and, similarly, showed the lowest frequency (< 20%) of highly infected individuals (Fig. 4). However, in colony 5 and 6, most individual bees were non-infected (53–75%; Fig. 4) and yet the mean spores per bee based on pooled sampling (Fig. 3) was above the treatment threshold of 1 × 10^6^ spores/bee.

### Comparison of *Vairimorpha* species prevalence in pooled 60- and 180-bee samples

The species prevalence did not differ significantly between samples of 60 or 180 bees from each colony (W = 0.8798, *p* = 0.426), nor among the treatment groups (F_(2,12)_ = 0.046, *p* = 0.955) or the combination of treatment and number of bees per pooled sample (F_(2,12)_ = 0.014, *p* = 0.986; Fig. 5). The *Vairimorpha* spp. ratio was 61.9–99.9% *V. apis* to 0.01–38.1% *V. ceranae*; the trend toward increased *V. apis* prevalence with increased mean spores per bee based on pooled samples was nonsignificant (*r* = 0.3835, *p* = 0.0781). Moreover, an increasing percentage of non-infected bees per colony (Fig. 4) was correlated significantly with decreasing *V. apis* prevalence (*r* = –0.8044, *p* = 0.0089).

## Discussion

We aimed to determine the effect of different sampling methods on the estimation of *Vairimorpha* spp. spore loads and species ratio at the colony level. The mean *Vairimorpha* spp. spore count in a sample of 60 pooled bees did not differ significantly compared to samples containing 120 and 180 pooled bees, indicating that increasing the number of pooled bees by 2–3-fold will not yield a more accurate estimate of colony infection. Interestingly, 2 colonies with mean spore counts per bee in excess of the treatment threshold of 1 × 10^6^ spores/bee consisted of a majority of non-infected or low-infected bees, which confirms our hypothesis that a low number of high-infected bees in a pool of healthy bees can lead to misinterpretation of the colony infection level and hence to antibiotic treatment of mainly uninfected bees. It is unclear if these low numbers of heavily infected nestmates would altruistically leave or would be forced out of the colony by healthy nestmates to maintain the colony health, as shown in previous studies.^1,5,24^

The number of pooled bees does not appear to influence the *Vairimorpha* spp. composition, as evidenced by the similar species prevalence in sample sizes of 60 or 180 pooled bees. Although we inoculated the colonies with a 50% mix of each *Vairimorpha* spp., surprisingly all colonies were predominantly *V. apis*–infected in spring (90% *V. apis* in 7 of 9 colonies). Recent literature suggested a replacement of *V. apis* by *V. ceranae*, which is contradictory to our findings.^8,15,22^ One explanation for this discrepancy is that *V. ceranae* spores in our experiment may have lost viability during the inoculum preparation, resulting in a higher *V. apis* prevalence in spring. Hence, our results are not in concordance with the occurrence of *V. ceranae* spring peaks shown for overwintering colonies in Ontario, Canada.^9^ Interestingly, colonies 5 and 6, which had mainly uninfected bees, revealed a higher *V. ceranae* proportion (~32%) than the other colonies (< 10%).

Our study highlights the difficulties of conducting experimental, colony-level infection with *Vairimorpha* spp., including high colony mortality and contamination of control colonies. Given the high overwinter mortality of our experimental colonies, we could sample only 10 of 21 colonies in spring 2022. Furthermore, spring *Vairimorpha* spp. infection levels in the control colonies were similar to the low- and high-dose colonies. Given the lack of protocols and studies using experimental *Vairimorpha* spp. infection at the colony level, we may have used exceedingly high spore doses for inoculation, ultimately resulting in high colony mortality. Also, the proximity of control colonies to *Vairimorpha* spp.–inoculated colonies in the same apiary may have resulted in *Vairimorpha* spp. transmission to control colonies through robbing of contaminated honey stores in dead or weak low- and high-dose colonies.^19^

Hence, we need further colony-level studies with higher sample sizes and non-contaminated controls to investigate the clinical importance of the frequency of *Vairimorpha* spp. infection intensity in individual bees. Our data will subsequently contribute to evaluation of antibiotic treatment strategies and the effect of treatment in colonies with a low frequency of highly infected individuals.

## Supplemental Material

sj-xlsx-1-jvd-10.1177_10406387231194620 – Supplemental material for Comparison of individual and pooled sampling methods for estimation of Vairimorpha (Nosema) spp. levels in experimentally infected honey bee colonies Click here for additional data file. Supplemental material, sj-xlsx-1-jvd-10.1177_10406387231194620 for Comparison of individual and pooled sampling methods for estimation of Vairimorpha (Nosema) spp. levels in experimentally infected honey bee colonies by Sarah Biganski, Tessa Lester, Oleksii Obshta, Midhun S. Jose, Jenna M. Thebeau, Fatima Masood, Marina C. B. Silva, Marcelo P. Camilli, Muhammad F. Raza, Michael W. Zabrodski, Ivanna Kozii, Roman Koziy, Igor Moshynskyy, Elemir Simko and Sarah C. Wood in Journal of Veterinary Diagnostic Investigation
